# Conocimiento de la enfermedad pulmonar obstructiva crónica (EPOC) en la población gallega: estudio «CoñecEPOC»

**DOI:** 10.1016/j.opresp.2021.100104

**Published:** 2021-05-12

**Authors:** Cristina Represas-Represas, Rafael Golpe-Gómez, Pedro J. Marcos-Rodríguez, Alberto Fernández-García, María Torres-Durán, Mónica Pérez-Ríos, Alberto Ruano-Raviña, Alberto Fernández-Villar

**Affiliations:** a Servicio de Neumología, Hospital Álvaro Cunqueiro, Vigo, Pontevedra, España; b Grupo de investigación NeumoVigo I + i, Instituto de Investigación Sanitaria Galicia Sur (IISGS), Galicia, España; c Servicio de Neumología, Hospital Universitario Lucus Augusti, Lugo, España; d Servicio de Neumología, Dirección Asistencial, Complejo Hospitalario Universitario de A Coruña (CHUAC), Instituto de Investigación Biomédica de A Coruña (INIBIC), A Coruña, España; e Área de Medicina Preventiva y Salud Pública, Universidad de Santiago de Compostela, Santiago de Compostela, España; f CIBER de Epidemiología y Salud Pública (CIBERESP), Madrid, España

**Keywords:** EPOC, Conocimiento, Espirometría, Población, Galicia, Estudios, COPD, Knowledge, Spirometry, Population, Galician, Studies

## Abstract

**Objetivos:**

En el año 2011, solo el 18% de la población en Galicia sabía qué es la EPOC. Desde entonces, se han realizado actividades para dar a conocer esta enfermedad. El objetivo de este estudio fue evaluar la situación actual sobre el conocimiento de la EPOC en población gallega.

**Metodología:**

Estudio transversal, mediante encuestas telefónicas. Se analizan variables incluidas en un cuestionario específico, relacionadas con el conocimiento de la enfermedad.

**Resultados:**

Fueron 872 encuestados, 53% mujeres, edad media 54 años. Un 63% con estudios secundarios/universitarios. El 40% sabe qué es la EPOC. En cambio, más del 90% de los encuestados conocen otras enfermedades de alta frecuencia (diabetes, ictus, asma). Los factores más asociados con el conocimiento de la EPOC fueron el género femenino, tener estudios secundarios/universitarios y haber realizado previamente una espirometría.

**Conclusiones:**

El conocimiento actual de la EPOC en población gallega es del 40%, superior al del año 2011, pero todavía está lejos del de otras enfermedades prevalentes.

## Abstract (en)

**Objectives:**

In 2011, only 18% of the population in Galician knew the COPD. Since then, activities have been carried out to publicize this disease. The objective of this study was to evaluate the current situation regarding the knowledge of COPD in the Galician population.

**Methodology:**

Cross-sectional study, through telephone surveys. Variables included in the questionnaire, related to knowledge of the disease, were analyzed.

**Results:**

872 respondents, 53% women, mean age 54 years. 63% with secondary/university studies. 40% has knowledge of COPD. In contrast, more than 90% of respondents know other high-frequency diseases (diabetes, stroke, asthma). The factors most associated with knowledge of COPD were female gender, having secondary/university studies, and having previously performed spirometry.

**Conclusions:**

The knowledge of COPD in the Galician population is 40% now, higher than in 2011, but it is far from that of other prevalent diseases.

## Introducción

Una de las causas del elevado infradiagnóstico de la EPOC en nuestro país –74,7% actualmente según EPISCAN II^1^–, es el desconocimiento de las características de la enfermedad por la población general^2^. Esta falta de conocimiento de la EPOC por la población española se ha demostrado en varios estudios. Uno de ellos es el estudio CONOCEPOC del 2011^3^, en el que se encuestó telefónicamente a 6.528 personas de ≥40 años de todo el territorio nacional empleando un sistema de entrevistas telefónicas asistidas por ordenador, observando que solo el 17% conocían la EPOC. Comparados estos datos con los de un estudio similar del año 2002, el conocimiento de la enfermedad se había duplicado (8,6% en ese momento^4^, pero lejos aún de lo deseable. En CONOCEPOC del 2011 se aportan datos por comunidades autónomas, y el conocimiento en Galicia se situaba en el 17,9%, similar a la media nacional^3^.

A lo largo de los últimos años, desde el grupo gallego de EPOC de la Sociedad Gallega de Patología Respiratoria se han llevado a cabo actividades divulgativas sobre esta enfermedad para pacientes y población general, con presencia en prensa, televisiones locales y redes sociales, así como el diseño de programas validados para generalizar el uso de la espirometría en Atención Primaria^5^. Es importante evaluar si esto ha podido contribuir a un mejor conocimiento sobre la EPOC en nuestra comunidad, y por esto se realizó este estudio.

## Metodología

### Diseño

Estudio transversal, basado en la realización de encuestas telefónicas asistidas por ordenador utilizando bases de datos de teléfonos fijos y móviles, generados por procedimiento de dígitos aleatorios asistidos por lista truncada. La población objetivo fueron mayores de 18 años de las 4 provincias gallegas, con muestras estratificadas por población de cada provincia (41,6% A Coruña, 12,7% Lugo, 11,6% Orense, 34,1% Pontevedra), tamaño del municipio de residencia (ámbito rural o urbano si menor o mayor de 10.000 habitantes), sexo y edad (rangos 18-30, 31-40, 41-50, 51-60, 61-70, >70 años), que aceptaban voluntariamente responder a un cuestionario por vía telefónica, de forma anónima y confidencial. El trabajo de campo fue realizado por la empresa especializada Instituto Sondaxe, con encuestadores capacitados y entrenados, durante el mes de octubre de 2019.

### Recogida de información

Tras la aceptación voluntaria del participante, se recogió la información de variables incluidas en un cuestionario diseñado *ad hoc*: edad, sexo, ámbito de residencia (rural o urbano), nivel de estudios (sin estudios, primarios, secundarios, universitarios), tabaquismo, presencia de síntomas respiratorios y si consultó por ellos, padecer alguna enfermedad respiratoria, conocimiento espontáneo e inducido de la EPOC, medio a través del cual la conoce, conocimiento de otras enfermedades prevalentes (asma, diabetes, ictus), y si había realizado una espirometría previamente. De forma similar a estudios previos^3^, el conocimiento espontáneo se evaluó al preguntar si sabe qué es la EPOC, el concepto de la enfermedad, sin ninguna orientación por parte del entrevistador, y se les preguntaba además que nombraran los síntomas con los que la relacionaban. A los que no la conocían, se le dio alguna orientación («enfermedad producida por el tabaco, provoca tos, flemas, fatiga…») y se les volvía a preguntar si así la conocen (conocimiento inducido).

### Análisis estadístico

Se realizó un análisis descriptivo, mostrándose las variables cualitativas como frecuencias y porcentajes, y las cuantitativas como medias y desviación estándar. Se llevó a cabo un análisis comparativo de variables mediante la prueba de chi-cuadrado y la t de Student, entre los sujetos que sí conocían la EPOC de forma espontánea y los que no. A continuación, se realizó un análisis de regresión logística adelante condicional en el cual se incluyeron todas las variables que en el estudio bivariante presentaron una p < 0,10, siendo la variable dependiente el conocimiento espontáneo de la EPOC, calculando las odss ratio (OR) con su intervalo de confianza del 95% (IC95%). En todas las comparaciones se consideraron diferencias significativas los valores de p inferiores a 0,05.

### Aspectos éticos

Los investigadores respetaron los principios de la Declaración de Helsinki, y aseguraron el anonimato de los participantes. Antes de pasar el cuestionario en la llamada telefónica, se informó al participante sobre el motivo del estudio y por qué se le invitaba a participar. Se consideró que el sujeto consiente participar si responde a las preguntas de forma voluntaria. El manejo de los datos del estudio, así como la base de datos elaborada, cumplirán los requisitos marcados en el Reglamento (UE) 2016/679 y del Parlamento Europeo y del Consejo del 27 de abril de 2016, relativo a la protección de las personas físicas en lo que respecta al tratamiento de datos personales y a la libre circulación de datos, y con la Ley Orgánica 3/2018, de 5 de diciembre, de Protección de Datos Personales y garantía de los derechos digitales. Todos los datos se almacenaron de forma segura y solo son accesibles para los miembros del equipo de investigación y personal autorizado. Al tratarse de una encuesta telefónica anónima y voluntaria, donde el participante da su consentimiento verbal para responder, este tipo de estudio no requiere la aprobación por el comité de ética.

## Resultados

Participaron 872 personas, tras realizar 4.989 llamadas, con cuotas representativas por rango de edad y provincia. En la tabla 1 se muestran las características de la muestra global, así como su comparación entre los que conocían la EPOC y los que no. La frecuencia de tabaquismo activo era mayor en hombres (30%) que en mujeres (19%). El 10,7% de la población (n = 93) referían padecer alguna enfermedad respiratoria crónica (la más frecuente asma, 8,7% del total; solo 1,9% EPOC). El 45,8% (n = 399) realizaron alguna vez una espirometría; 156 (17,9%) referían algún síntoma respiratorio, pero solo la mitad consultaron por ellos.

**Tabla 1:** Características de la población encuestada, y análisis comparativo según conocimiento espontáneo de la EPOC de los estudios CONOCEPOC-2019 y CoñecEPOC, y diferencias entre los que sí conocen la EPOC y los que no la conocen en CoñecEPOC

	CONOCEPOC 2019	CoñecEPOC			
	Muestra global n = 1920	Muestra global n = 872	Sí conocen n = 350	No conocen n = 522	p
Edad, años*,^a^	61,9 (13,5)	54 (19)	52 (17)	55 (20)	0,04
Sexo femenino^a^	1313 (68%)	463 (53%)	198 (56%)	265 (51%)	0,09
Tabaquismo activo^a^	279 (14,5%)	212 (24%)	105 (30%)	107 (21%)	0,01
Consumo acumulado tabaco*^a^	21 (22,7)	14,5 (15)	16,7 (16)	12,5 (13)	0,04
Estudios secundarios/universitarios	NA	547 (63%)	262 (75%)	285 (55%)	< 0,01
Hábitat urbano	NA	607 (69,6%)	244 (69,7%)	363 (69,5%)	0,95
Realización de espirometría previa^a^	139 (7,2%)	399 (45,8%)	186 (53,1%)	213 (41%)	< 0,01
Síntomas respiratorios referidos^b^	354 (18,4%)	156 (17,9%)	78 (22,3%)	78 (14,9%)	< 0,01

* Expresado como media (desviación estándar).

a p < 0,0001 en la comparación de la variable de las muestras globales de CONOCEPOC-2019 vs. CoñeceEPOC.

b p 0,72 en la comparación de la variable de las muestras globales de CONOCEPOC-2019 vs. CoñecEPOC. NA: no analizado.

El 40% de la población encuestada sabe lo que es la EPOC (conocimiento espontáneo), sin diferencias por provincia (40,5% A Coruña, 40,5% Lugo, 39,6% Pontevedra, 39,7% Orense). La mayoría de los participantes referían conocerla por los medios de comunicación (35%), un 32% por tener alguna persona cercana con la enfermedad, y solo un 17% a través de información recibida por profesionales sanitarios. Además, el conocimiento inducido fue del 32%. El 98% de los encuestados sabía qué es la diabetes, el 91% el ictus, y el 97% el asma. Las variables que mostraron diferencias significativas entre los sujetos que sí conocían la EPOC de forma espontánea y los que no fueron la edad, el tabaquismo, el nivel de estudios, la realización previa de una espirometría y la presencia de síntomas respiratorios (tabla 1).

En la tabla 2 se muestran los datos de conocimiento espontáneo en base a los rangos de edad, oscilando entre el 26,3% en los mayores de 70 años y el 54,4% entre los de 51 a 60 años.

**Tabla 2:** Conocimiento espontáneo en estudios CoñecEPOC y CONOCEPOC-2019 según rango de edad

Edad	CoñecEPOC	CONOCEPOC 2019	p
18 – 30 años	40/112 (35,7%)	NA	-
31 – 40 años	55/130 (42,3%)	NA	-
40 – 50 años	68/164 (41,5%)	142/480 (29,5%)	0,005
51 – 60 años	84/154 (54,5%)	145/480 (30,2%)	0,00001
61 – 70 años	54/126 (42,8%)	150/480 (31,2%)	0,001
> 70 años	49/186 (26,3%)	98/480 (20,4%)	0,09

NA: no analizado.

En el análisis multivariante, los factores que se relacionaron de forma independiente con este conocimiento de la EPOC fueron: el sexo femenino [OR 1,5 (IC95% 1,1-2)]; tener estudios secundarios o universitarios [OR 2,1 (IC95% 1,4-3)]; y haber realizado previamente una espirometría [OR 2 (IC95%1,4-2,7)].

## Discusión

El 40% de la población gallega sabe qué es la EPOC (conocimiento espontáneo). Sin embargo, a pesar de ser una de las enfermedades más prevalentes, este dato contrasta negativamente con el conocimiento de otras patologías muy frecuentes, ya que casi la totalidad de la población sabe qué es la diabetes (98%), el ictus (91%) o el asma (97%).

Este porcentaje es significativamente superior al observado para Galicia en el estudio CONOCEPOC de 2011, y al conocimiento global en el recientemente publicado CONOCEPOC-2019^6^*,* con metodología similar al de 2011 pero menor tamaño muestral (1.920 personas), y con 139 (7,2%) participantes gallegos, pero en el que no se publicó análisis de datos por comunidades. En este caso el 27,9% de la población española ≥40 años tenía conocimiento espontáneo de la EPOC. Los factores que se mostraron como predictores independientes de conocimiento de esta enfermedad en nuestro estudio fueron el sexo femenino, tener estudios secundarios o universitarios y el haber realizado previamente una espirometría (este último, además de la presencia de síntomas respiratorios, predictor también en CONOCEPOC-2019). Nos congratularía mucho pensar que las actividades llevadas a cabo en la comunidad gallega, impulsadas por el grupo gallego de EPOC de la Sociedad Gallega de Patología Respiratoria, son las responsables de este mayor nivel de conocimiento de la EPOC respecto a la media nacional. Pero quizás las razones que expliquen esto sean otras. Como se puede observar en la tabla comparativa (tabla 1) las poblaciones de estudio muestran claras diferencias. En CoñecEPOC la edad media es menor porque se incluyeron sujetos a partir de los 18 años (≥40 en CONOCEPOC-2019), pero con la fortaleza de que se estratificaron por edad, sexo (en CONOCEPOC-2019 tienen un mayor % de mujeres), ámbito y provincia. De hecho, el haber introducido una edad menor en CoñecEPOC, podría significar un menor conocimiento global asociado a una menor prevalencia de la enfermedad, y el resultado observado es el opuesto. Además, si comparamos los porcentajes de conocimiento espontáneo entre los mismos rangos de edad de los dos estudios (tabla 2), se sigue mostrando un mayor grado de conocimiento en el estudio gallego en todos los rangos de edad comparables salvo en el grupo de mayores de 70 años en el que, aunque el porcentaje que sí conocen la EPOC es superior en CoñecEPOC, no se alcanzan diferencias estadísticamente significativas. En CoñecEPOC un alto porcentaje habían cursado estudios secundarios o universitarios, aspecto que además se demostró como predictor independiente de conocimiento de la enfermedad, mientras que el nivel de estudios no se ha recogido en CONOCEPOC-2019, y este podría ser un factor clave. El otro predictor de conocimiento espontáneo de la EPOC fue la realización previa de una espirometría y este porcentaje también fue claramente superior en CoñecEPOC (45,8% vs*.* 9,6%). Por tanto, podría ser un tema a debate si las diferencias de los resultados entre CoñecEPOC y CONOCEPOC-2019^6^, se deben a las distintas características de las poblaciones evaluadas o por pura variabilidad interregional.

Cabe destacar que en el grupo de los que no conocen la EPOC, un 21% son fumadores activos, lo que vuelve a poner de relieve el desconocimiento de los efectos del tabaquismo a nivel respiratorio incluso entre los propios fumadores. También es un dato a resaltar que solo el 17% de los que conocen la EPOC lo han hecho a través de profesionales sanitarios, poniendo de manifiesto que nuestra función divulgativa está un tanto olvidada, y debe ser retomada, como un pilar igual de importante que la asistencia, la docencia o la investigación.

A modo de conclusión, el estudio CoñecEPOC nos muestra que el 40% de la población general gallega saben qué es la EPOC, y por tanto este conocimiento es superior al del año 2011 en esta misma comunidad, y superando también a los datos a nivel nacional. Sin embargo, este conocimiento queda todavía lejos del de otras enfermedades, por lo que debemos seguir trabajando para una mayor concienciación en la población sobre la importancia de las enfermedades respiratorias, fundamentalmente en aquellas asociadas al tabaco, como la EPOC.

## Financiación

La realización del presente estudio ha sido posible gracias a una ayuda no condicionada de Boehringer-Ingelheim.

## Autoría/colaboradores

Concepción y diseño del estudio: CRR, RGG, JPMR, MTD, AFV.

Recogida, análisis e interpretación de datos: CRR, RGG, JPMR, AFG, AFV, ARR, MPR.

Escritura del artículo y revisión crítica: CRR, AFV, ARR, MPR.

Aprobación final del artículo: todos los autores.

## Conflicto de intereses

Los autores declaran no tener ningún conflicto de intereses.
